# Theory of Mind: A Brief Review of Candidate Genes

**DOI:** 10.3390/genes15060717

**Published:** 2024-05-31

**Authors:** Corrado Silvestri, Simona Scaini, Ludovica Giani, Mattia Ferro, Maria Nobile, Marcella Caputi

**Affiliations:** 1 Child and Youth Lab, Sigmund Freud University of Milan, Via Ripa di Porta Ticinese 77, 20143 Milan, Italy; corrado.silvestri01@gmail.com (C.S.); giani.phd@milano-sfu.it (L.G.); 2 Child and Adolescent Unit, Italian Psychotherapy Clinics, Corso San Gottardo 5, 20136 Milan, Italy; 3 Brain and Behaviour Lab, Sigmund Freud University of Milan, Via Ripa di Porta Ticinese 77, 20143 Milan, Italy; m.ferro@milano-sfu.it; 4 Child Psychopathology Unit, Scientific Institute, IRCCS Eugenio Medea, Bosisio Parini, 23842 Lecco, Italy; maria.nobile4@gmail.com; 5 Department of Life Sciences, University of Trieste, Via E. Weiss, 34128 Trieste, Italy; marcella.caputi@units.it

**Keywords:** theory of mind, mentalization, genes polymorphism, genetic basis

## Abstract

Deficits in theory of mind (ToM), known as the ability to understand the other’s mind, have been associated with several psychopathological outcomes. The present systematic review aims to summarize the results of genetic studies that investigated gene polymorphisms associated with mentalization performance tasks in children and adults. The systematic review was carried out following PRISMA guidelines, and the literature search was conducted in PubMed and EBSCOhost using the following keywords: ‘theory of mind, mentalizing, mindreading’ and ‘gene, genetic basis’. Nineteen studies met the eligibility criteria for inclusion. Most of the literature focused on the role of *DRD4*, *DAT1*, *OXTR*, *OXT*, *COMT*, *ZNF804A*, *AVP*, *AVPR*, *SCL6A4*, *EFHC2*, *MAO-A*, and the family of *GTF2I* genes in influencing ToM. However, controversial results emerged in sustaining the link between specific genetic polymorphisms and mentalization abilities in children and adults. Available data show heterogeneous outcomes, with studies reporting an association between the same family genes in subjects of the same age and other studies reporting no correlation. This does not allow us to draw any solid conclusions but paves the way for exploring genes involved in ToM tasks.

## 1. Introduction

Theory of mind (ToM) is a sociocognitive ability that allows people to infer their own and others’ mental states [1,2,3,4,5], using them to decipher and predict behaviors [6]. Similarly, mentalizing refers to the ability to understand and interpret one’s own and others’ actions as being intentional and goal-directed but involves a reflective process where one actively considers and makes sense of the self–other distinction and the subjective nature of mental experiences [7]. These two terms both describe metacognitive processes and are often used interchangeably [8], but slight differences subsist. In particular, mentalization indicates an activity of reflection on affective mental states, whereas ToM refers to epistemic states, such as beliefs and intentions.

Deficits in the correct attribution of other mental states are present in several psychopathologies, with the most studied condition being represented by the autism spectrum disorder (ASD) [9,10]. It is widely acknowledged in the context of ASD that individuals often lack a functional theory of mind (ToM) [11]; specifically, Baron-Cohen’s research shows [12] people diagnosed with autism exhibit the ability to decipher observed actions by understanding their underlying goals and desires [12]. They can also interpret sensory input based on the perspective of an agent [12]. In addition, they encounter a significant deficiency in what he terms the Shared-Attention Mechanism, a system that deals with triadic relationships involving oneself, the other’s mind, and an object of interest, commonly known as joint attention; furthermore, individuals with ASD face challenges in grasping the propositional attitudes of others [12]. This holds true not only for individuals with ASD in general but also extends to adults with high-functioning autism [13]. It is also thought that a deficit in ToM might contribute to the lack of social skills and communication shown in the autistic spectrum [11]. This ability can be severely compromised in a wide variety of other psychological disorders [9,14]. Specifically, two personality disorders seem to have a strong impairment at the mentalization level: antisocial personality disorder (ASPD) and borderline personality disorder (BPD) [7].

Considering the strong impairment in the mentalistic abilities of patients with borderline [15] and antisocial personality, an evidence-based therapeutic intervention program has been developed, aimed at restoring the ability to mentalize [16]. This program is called Mentalization-Based Treatment (MBT) [7,17,18]. For the patient, the goal is to discover more about how they think and perceive themselves and others, how this influences their way of relating, and how some errors in self and other understanding can lead to misunderstanding, dysfunctional behaviors, as well as emotional instability [7]. This type of treatment has proven to be effective for BPD; compared to standard treatments, MBT seems to help the reduction in suicidality and access to services [19]. In adolescents, it has shown a recovery rate of 44%, compared to the 17% of standard treatment [20]. As for ASPD, MBT appears to assess the severity of progressively decreasing aggressions and has led to a decrease in depressive and anxious symptoms [21].

People affected by schizophrenia and neurodegenerative disorders, such as frontotemporal dementia [22], Parkinson’s disease [23], and Alzheimer’s disease [22], also show severe ToM deficits [24,25,26,27,28]. Specifically, misunderstandings regarding the mental states of others often arise from challenges in effectively monitoring one’s own and others’ behavior and mental conditions, as indicated by the literature [29,30,31]. This is further compounded by a difficulty in integrating relevant contextual information [32]. Additionally, there is a potential phenomenon referred to as “Hyper ToM”, wherein patients tend to excessively attribute intentions, desires, or emotions to others, as proposed by Abu-Akel and Shamay-Tsoory [24]. This impairment has been found to account for an estimated 15% to 40% of the variability in social functioning in individuals with schizophrenia, as indicated by several studies [26,33,34,35,36,37].

Among the children population, many disorders are associated with deficits in understanding other’s minds. Fetal alcohol syndrome (FAS) is one of the numerous disorders in which researchers have found an association with mentalizing problems [38,39]. Children with oppositional defiant disorder (ODD) and conduct disorder (CD) also seem to show difficulties in understanding and processing social information [40,41]. Focusing on neurodevelopmental disorders (NDDs), it is possible to find many impairments within this type of diagnostic category. Children with attention deficit hyperactivity disorder (ADHD) show low performance on theory of mind tasks compared with a healthy group [42,43], probably due to an impairment of executive functions [42], as shown in a recent review [44]. Deficits in ToM abilities are also encountered among children with specific learning disorders [45,46] and children with intellectual disabilities [47,48]. There is evidence that ToM deficits have been detected in highly hereditary diseases, such as the 22q11.2 deletion syndrome [49], Down syndrome [50], Martin Bell syndrome [50], Phenylketonuria (PKU) [51], Prader–Willi syndrome [52], cerebrospinal ataxia [53], Turner syndrome [54], and Williams syndrome [55]. In light of these considerations, it is plausible to hypothesize that the capacity for mentalization has a genetic foundation.

The wide spectrum of psychopathologies encountering ToM deficits in their phenotype has paved the way for better understanding its etiopathogenesis, which requires a comprehensive assessment comparing the affected population with the healthy one. In line with the biopsychosocial model [56] of modern psychiatry, the etiology of each disorder is given by the interaction between several risk factors, both genetic and environmental ones, each accounting for specific quotes of variance of the investigated behavioral phenotypes. Therefore, the presence of biological latent vulnerabilities not triggered by the exposition to environmental factors is not expected to turn into a psychopathological outcome. However, several types of environmental risks and variables could lead to an impairment of the ability to understand the minds of others. Being adopted after early institution [57,58] and growing up in physically abusive and neglecting families [59] figure among the most prominent environmental risk factors for ToM.

With regard to the genetic risk factors involved in the development of ToM abilities, the literature has focused on well-established genetically determined conditions with mentalization deficits, like the 22q11.2 deletion syndrome [60], Down syndrome [61], Martin Bell syndrome [61], Phenylketonuria (PKU) [62], Prader–Willi syndrome [63], cerebrospinal ataxia [64], Turner syndrome [65], and Williams syndrome [66], to detect candidate genes, supporting in this way the hypothesis that ToM has a genetic basis [49,50,67] and the innate and modular vision of this ability proposed by several authors [51,52,53,54,55,68]. However, little is known about the potential polymorphisms and genetic candidates involved in the etiopathogenesis of ToM.

In light of these premises, it seems reasonable to assume that the ability to mentalize has a genetic basis. Therefore, the present systematic review is aimed at summarizing the results of genetic studies that have investigated theory of mind/mentalization tasks with polymorphism genes in healthy children and adults, clarifying the role of genes in the ability to understand others’ minds. This review considers all articles published up to February 2024.

## 2. Materials and Methods

The present systematic review was carried out following the PRISMA 2020 standards on two search engines, PubMed and EBSCOhost. The following keywords were used in the research process: ‘theory of mind, mentalizing, mindreading’ and ‘gene, genetic basis’. The following inclusion criteria have been applied to the research: (a) published English-language papers; (b) studies carrying out a genotyping process and investigating theory of mind/mentalization through specific tasks; and (c) studies on atypical populations only if a healthy control group was present. Studies that included only atypical populations were excluded, as well as books, theses, systematic reviews, and meta-analyses. The research did not consider a specific timeframe. In total, the search produced 246 articles, 100 on PubMed and 146 on EBSCOhost.

**Figure 1:**
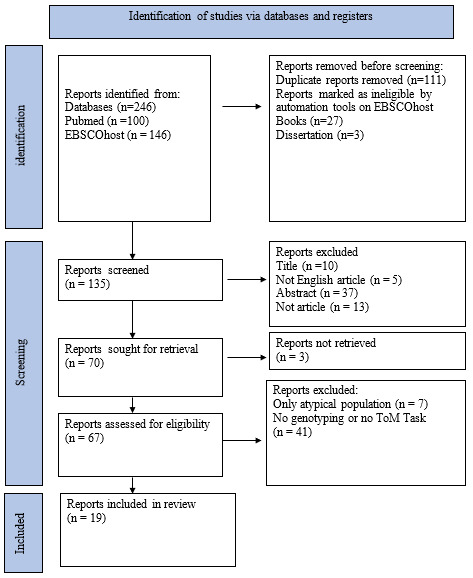
Flow chart.

The authors screened the search results eliminating a priori, only for the EBSCOhost’s results, books, and theses via the automatic tool option of the website; subsequently, the duplicates were manually removed, bringing out a total of 146 items. These articles were filtered manually, removing irrelevant articles based on title, abstract, article type, and article language. The next step was to filter the additional articles manually, excluding papers that did not meet the inclusion criteria. Nineteen was the total number of studies that met the eligibility criteria for the present systematic review (see Figure 1).

## 3. Results

The research produced 19 studies that examined several genes involved in the etiopathogenesis of mind theory skills: *DRD4*, *DAT1*, *OXTR*, *OXT*, *COMT*, *ZNF804A*, *AVP*, *AVPR*, *SCL6A4*, *EFHC2*, *MAO-A*, and the *GTF2I* gene family.

The dopamine D4 receptor gene (*DRD4*) [69,70,71,72,73]; the dopamine reuptake transporter polymorphisms in the 3′ UTR (*DAT1*) [70,72,73]; Zinc-finger protein gene polymorphism rs1344706 (*ZNF804A*) [74,75]; the catechol-O-methyltransferase enzyme gene polymorphism Val158Met (*COMT*) [70,71,73,76]; the serotonin carrier gene polymorphism 5-HTTLPR (*SCL6A4*) [73,77]; the oxytocin receptor gene polymorphisms (*OXTR*) and the oxytocin gene polymorphisms (*OXT*) [71,78,79,80,81,82,83]; the arginine vasopressin hormonal genes polymorphisms (*AVP*) and the arginine vasopressin subtype 1A receptor gene polymorphisms (*AVPR1A*) [79,84]; other polymorphisms including the ones belonging to *MAO-A* genes, *EFHC2* genes, and *GTF2I* genes [85,86,87].

The selected studies are summarized in Table 1. Of these 19 studies, eight recruited a sample of children [69,70,71,78,79,80,84,87], ten a sample of adults [72,73,74,75,76,77,81,83,85,86], and one a sample of adults and teenagers [82].

### 3.1. Genes

#### 3.1.1. *DRD4* Gene

Focusing on the dopamine D4 receptor gene (*DRD4*), one study [69] found that, for children, there was an association between the *DRD4-III 7R* allele gene and affective knowledge (ability to attribute emotional mental states, facial expressions, and correspondence between these two) [69] in interaction with gender [Wald χ2(1, N = 280) = 7.66, *p* = 0.006]. In particular, among *7R*-allele carriers, males scored significantly higher than females [Wald χ2(1, N = 283) = 4.238, *p* = 0.04], meaning that they have a better ability to understand the mind of others, while for the noncarriers of the 7R allele, there was no significant gender effect [Wald χ2(1, N = 283) = 0.28, *p* = 0.56]. As far as girls are concerned, the bearers of the 7R allele were associated with lower performance than the non-carriers [Wald χ2(1, N = 280) = 5.85, *p* = 0.016]; the latter, therefore, show higher performance in the task. Another study [70] on children shows that there is an association between the *DRD4* gene polymorphisms and the representational theory of mind (RTM); particularly, the group with at least one long allele (≥6 repetitions) showed worse performance in the RTM battery [88] than the group that had two short alleles (≤4 repetitions) [F(1, 68) = 8.19, *p* = 0.006, η2 = 0.107]. The last study [71] on children for this gene proved there was no significant association between the allelic variations of *DRD4* VNTR 48 bp and the theory of mind [*p* = 0.810; *p* = 0.680; *p* = 0.327]. Concerning the adult sample, two studies were considered; one [73] showed that in the healthy population, there was no association between the Reading the Mind in the Eyes task [89] and the genotype [Wilks’ λ = 0.92, F(2, 50) = 2.29, *p* = 0.11, η2 = 0.08]. The other study [72] did not show any type of polymorphism with a significant effect in the healthy sample.

#### 3.1.2. *DAT1* Gene

Three studies explored the relationship between the dopamine reuptake transporter polymorphisms in the 3′ UTR *(DAT1)* and the theory of mind. One study [70] conducted on children found that there was no association between the representational theory of mind [88] and genotype controlling by age [70]. For adults, Tadmor and colleagues [72] found that the group with a 9/9 genotype, among both schizophrenics and healthy groups, showed a worse tendency in the scores of Reading the Mind in the Eyes [89] than 10/10 and 9/10 polymorphisms [β = −0.15, t = −2.1, *p* = 0.04]. The second study conducted on adults [73] showed that, after dividing the components of the study into variant 9 carriers (9/9 and 9/10) and variant 10/10 carriers, no significant association was found between the healthy subjects [Wilks’ λ = 0.95, F(2, 51) = 1.39, *p* = 0.26, η2 = 0.05].

#### 3.1.3. *ZNF804A* Gene

Two studies have focused their attention on the relationship between the zinc-finger protein gene (*ZNF804A*) polymorphism rs1344706 and the theory of mind in adults. One study [74] shows that there is no difference, neither in the performance of the test nor in the times of reaction, between the carriers of the A allele and the non-bearers. The second study [75] demonstrates that there are no significant differences between the various genotyping groups with respect to both tests, the Hinting Tasks [29] and the Reading the Mind in the Eyes task [89] (Hinting task: F = 0.11, *p* = 0.89; Eyes task: F = 0.92, *p* = 0.40).

#### 3.1.4. *COMT* Gene

The role of *COMT* gene polymorphism Val158Met (rs4680) and the theory of mind was investigated by four studies. One study [70] shows that, controlling for age, there was no significant association between gene variants and the performance of the administered RTM battery in the children population. The other study [71] on children found the same result: there was no association between gene variations and the ToM ability. For the adult sample, things may look different, Xia and colleagues [76], after taking into account rs4680, rs4633, rs2020917, rs2239393, rs737865, rs174699, and rs59938883 polymorphisms, found that the rs2020917 polymorphism was associated with the cognitive ToM performance [F(2, 95) = 3.95, *p* = 0.023, g2 = 0.077], in particular carriers of the variant C/T showed better performances than C/C [*p* = 0.004] and T/T genotype [*p* = 0.043] [76]; the rs737865 polymorphism has been demonstrated to have also an association with the cognitive ToM [F(2, 95) = 3.90, *p* = 0.024, g2 = 0.076], and carriers of genotype C/C show higher scores than T/T group [*p* = 0.010] [76]. Regarding the affective component, it is noted that rs5993883 SNP showed a gender–genotype interaction [F(2, 95) = 3.35, *p* = 0.039, g2 = 0.066]; specifically, the males carrying the G/T variant have obtained lower performances than the females with the same genotype [*p* = 0.023], while women with T/T genotype showed higher scores of G/G carriers [*p* = 0.017] [76]. No significant association was found for the remaining polymorphisms [76]. For the last study on this gene [73], nothing significant was found by the authors [Wilks’ λ > 0.99, F(2, 89) = 0.16, *p* = 0.85, η2 = 0.004].

#### 3.1.5. *SCL6A4* Gene

Two studies focused on the 5-HTTLPR polymorphism of the *SCL6A4* gene. In the first study [73], researchers concluded that the score value was not significant between healthy subjects and the genotype [Wilks’ λ = 0.97, F(2, 55) = 0.79, *p* = 0.46, η2 = 0.03]. The other study [77] showed that carriers of low expression alleles (S/S; S/Lg; Lg/Lg) showed higher scores in recognizing negative mental states with increased exposure to negative life events [(b = −2.54, se = 1.21, t(210) = 2.10, *p* = 0.037; model r2 = 0.026)].

#### 3.1.6. *OXT* and *OXTR* Gene

There are many studies that focused on the role of the *OXTR* gene polymorphisms; four of them investigated the role during infancy and others on adults and teenagers. For children, one study [78] found out that, within rs1042778, rs2254298, rs11131149, rs237897, e rs237899 polymorphisms, there were no strong genetic effects of ToM; however, the analyses showed that there was an interaction between the gene and gender on the theory of mind, specifically on polymorphism rs11131149 [(zinter = 2.08, *p* = 0.04)]: women with larger G-allele copies showed better performance than men; furthermore, there was an interaction between rs11131149 SNP and maternal cognitive sensitivity [*p* = 0.019 major allele; *p* = 0.017 minor allele] where the latter predicted ToM ability in children. Researchers also found a mild interaction between haplotype formed by rs11131149-rs2254298 (G/A) and maternal cognitive sensitivity on the mind theory [*p* = 0.027] [78].

The research conducted by Wu and Su [80] demonstrated that, by checking for age, there was a significant effect between genotype and theory of mind [F(2,80) = 3.368, *p* = 0.039, η2 = 0.08]: those with the G/G genotype showed significantly higher scores than those who were carriers of the A/A genotype [(*p* = 0.032)] [80]. Other studies showed no significant interaction between children’s ToM and OXTR polymorphisms gene [71] or OXT polymorphisms gene and ToM [unadjusted model [*p* = 0.82, h2 < 0.001], adjusted model [*p* = 0.19, h = 0.003]] [79]. One study [81] showed an association between the OXTR gene SNP and ToM in adolescents; specifically, individuals carrying the C/C SNP rs2228485 genotype show lower scores in the Reading the Mind in the Eyes test [89] on male face test images than T genotype carriers, in both male and female groups [F(2) = 8.174, *p* = 0.000639]; Furthermore, significant scores were found for rs53576 SNP, where allele A carriers gave more correct answers in the required task [total faces: *p* = 0.022, female faces: *p* = 0.044], while for the rs2228485 SNP, the carriers of allele A identified with less frequency the images of positive matrix [*p* = 0.001205] [81]. Two studies concentrated on a sample of adults: one study found no significant association [82]; the same result was found by the second research [83], where no significant performance was found in the Hinting Task [29] and the OXTR rs53576. However, further analysis showed that high scores in schizotypy, assessed with SPQ-CP [90], are related to worse performance on ToM conditional to the G/G genotype [β = 0.468, *p* = 0.007, Radj2 = 30.1%].

#### 3.1.7. *AVP* and *AVPR1A* Gene

The role of *AVPR1A* and *AVP* gene polymorphisms was investigated, respectively, by Wade and colleagues [84] and Wade and colleagues [79]; for the first study [84], no association was found [rs1042615 *p* = 0.63; rs7298346 *p* = 0.74], while for the second study [79], researchers found a significant interaction between ToM scores and the haplotype *rs1887854-rs3761249* [unadjusted model (*p* = 0.0089; η2 = 0.021)].

#### 3.1.8. Other Genes

Other genes have been discovered to have a role in ToM; one study [85] found that between *MAO-A* gene polymorphisms, adults who had the low activity allele (2, 3, or 5 repetitions) showed significantly lower performance than those who had the high activity allele (3.5 or 4 repeated alleles) [F(1402) = 14.529, *p* = 0.00016, η2 = 0.035] [85]. Another study [86] revealed an association between the EF-hand domain containing 2 gene (*EFHC2*) polymorphism, *rs7055196*, and ToM scores in adults: males, with allele A, showed better performance than those who had allele G [t(89) = 2.04, *p* = 0.045, 95% CI (0.03, 2.40)]. The last study [87] investigated the role of the 1.1 Mb, 1.5 Mb, and 1.8 Mb deletions in *GTF2I* family genes; results showed that healthy subjects performed significantly better than other groups, suggesting that those who do not possess the *GTF2IRD1*, *GTF2I*, and *GTF2IRD2* genes, suffer from cognitive deficits such as the theory of mind [87].

**Table 1:** Selection of studies investigating genetic polymorphisms associated with theory of mind.

Study	Sample and Measures	Gene and Its Mutations	Main Findings
Ben-Israel et al., 2015 [69]	402 healthy children (161 children participated in both measurement) Mean age: first measurement (280 children): 44.13 ± 2.78 months second measurement (283 children): 61.73 ± 2.15 months Jerusalem Story Test of Interpersonal Understanding	*DRD4-III* gene: 7R allele carriers (presence at least one 7 repeat allele), 7R allele non-carriers (with 7 repeat allele absent)	7R-allele carriers males scored significantly higher than females in both age measurements. For 7R-allele non-carriers there was no effect of sex on affective knowledge. Girls with 7R allele were associated with lower performances than non-carriers. Examination of the genotype effect separately for boys and girls gave no significant results neither for boys nor for girls.
Lackner et al., 2012 [70]	73 healthy children Mean age 47.25 months Age range: 42–54 months RTM; NMR	*DRD4* gene: at least one long allele (≥ 6 repeats) both two shorts alleles (≤4 repeats). *COMT* gene Val/Met (rs4680) SNP: Val/Val; Val/Met; Met/Met. *DAT1* gene: 10/10; 10/9; 9⁄9	*DRD4* gene: the group with at least one long allele (≥6 repetitions) performed worse in the RTM battery than the group with two short alleles (≤4 repeats). *COMT* gene: controlling for age, no significant association between gene variants and ToM performance. DAT1 gene: researchers found that there was no association between the representational theory of mind and genotype controlling for age.
Opitz et al., 2021 [71]	80 healthy children Mean age: first measurement: 50 months (50.58 mean age ± 0.85) second measurement: 60 months (60.69 mean age ± 0.69) third measurement: 70 months (70.34 mean age ± 0.51) Wellman and Liu scale	*DRD4* gene: at least one long allele (≥ 6 repeats) all ≤5 repeat *COMT* rs4680 gene: A/A; A/G; G/G *OXTR* rs53576 gene: A/A; A/G; G/G	*DRD4* gene: the results show no significant association between allelic variations of DRD4 and theory of mind. *COMT* gene: there is no association between gene variations and ToM *OXTR* gene: there were no associations between genetic variants and theory of mind
Zahavi et al., 2016 [73]	96 students: 58 healthy students 38 with depression Age range: 18–30 years RMET	*DRD4* gene: ‘l-long’ (6–10 variants); ‘s-short’ (2–5 variants). *DAT1*: 9 variants (9/9 e 9/10); 10/10 variants. *COMT* gene: Val/Val; Val/Met; Met/Met. SCL6A4 gene, 5-HTTLPR SNP: at least one short allele (S/S or S/L); L/L genotype	*DRD4*: within healthy population there was no association between the value of photography and genotype. *DAT1*: no significant association was found in healthy subjects. *COMT* gene: no association was found; however, it was found that depressed subjects scored lower than the neutral tests compared to healthy subjects. *SCL6A4* gene, 5-HTTLPR SNP: the value of scores were not significant between healthy subjects and the genotype
Tadmor et al., 2016 [72]	270 subjects: 200 healthy subjects 70 with schizophrenia (SZ) age mean: 35.6 years ± 10.0 RMET	*DRD4* gene: long allele (≥5 repeats); short allele (≤4 repeats). *DAT1* gene: 10/10; 10/9; 9/9	*DRD4* gene: the healthy group showed better performance than the SZ group; however, no polymorphism was significant among the healthy sample. SZ with long allele decoded better the photographs with the positive valence than healthy individuals with the long allele; who was in the group SZ with short allele decoded less the photos with positive valence regarding the healthy group within the same genotype. *DAT1* gene: 9/9 genotype, for both groups schizophrenics and healthy, showed a worse tendency in ToM scores.
Walter et al., 2011 [74]	109 healthy subjects mean age: 32 years ToM condition (mentalizing) and a control condition (non-mentalizing) ToM comic strips	*ZNF804A* gene, rs1344706 SNP: C/C; C/A; A/A	*ZNF804A* gene, rs1344706 SNP: no difference neither on the reaction times of the test nor on the performance, between the carriers of the allele to risk (allele A) and the non-bearers.
Hargreaves et al., 2012 [75]	618 adults: 418 schizophrenic 200 healthy subjects Age range: 18–65 years HT; RMET	*ZNF804A* gene, rs1344706 SNP: C/C; C/A; A/A	*ZNF804A* gene, rs1344706 SNP: there are no significant differences between the various genotyping groups with respect to both tests
Xia et al., 2012 [76]	101 healthy adults Mean age: 22.50 years ± 2.28 affective ToM: white lie tasks; la faux pas tasks cognitive ToM: second-order false belief tasks; double bluff task.	*COMT* gene: rs4633, rs2020917, rs737865, rs174699 SNPs (diving the group for each polymorphism in C/C, T/T, C/T); rs4680 and rs2239393 SNPs (diving the group for each polymorphisms in A/A, A/G, G/G); rs59938883 SNP (diving the group for each polymorphisms in G/G, G/T e T/T).	*COMT* gene: cognitive ToM performance was associated with rs2020917 SNP, in particular, C/T carriers showed better performances than C/C genotype; rs737865 polymorphism was associated with cognitive ToM, C/C genotype carriers show higher scores than T/T group; there is a sex-genotype interaction between affective ToM and rs5993883 SNP, the G/T-carrying males have obtained lower performances than the females with the same genotype, while women with T/T genotype showed higher scores of G/G carriers; for the remaining SNP no significant association was found
Kruijt et al., 2014 [77]	215 healthy subjects Age range: 17 and 35 years RMET; LTE-Q	*SCL6A4* gene, 5-HTTLPR SNP: two low expressing alleles (SS; SLg; LgLg); one low and one high expressing allele (SLa; LgLa); high-expression allele (LaLa)	*SCL6A4* gene, 5-HTTLPR SNP: Carriers of low expression alleles (SS; SLg; LgLg) showed higher scores in recognizing negative mental states with increased exposure to negative life events assessed with LTE-Q.
Wade et al., 2015 [78]	301 healthy children Mean age: 4.79 years ± 0.28 Wellman and Liu scale	*OXTR* gene: rs1042778, rs2254298, rs11131149, rs237897, rs237899 SNPs	*OXTR* gene: there was an association between ToM and the rs11131149 SNP, in particular women with more copies of the G allele showed better performance than males; there is an interaction between rs11131149 SNP and maternal cognitive sensitivity, this covariate predicted ToM ability in children; there was an interaction between Haplotype rs11131149-rs2254298 (G/A) and maternal cognitive sensitivity on the theory of mind.
Wu & Su, 2015 [80]	87 children Age range: 3 to 5 years old Mean age: 4.5 years ± 9.03 months False-belief contents, False-belief location change task	*OXTR* gene, rs53576 SNP: A/A, A/G, G/G	*OXTR* gene, rs53576 SNP: by controlling for age, there was a significant effect between genotype and ToM, who possessed the genotype G/G showed scores significantly higher than those who were carriers of genotype A/A.
Wade et al., 2016 [79]	320 children Mean age: 4.79 years ± 0.28 Wellman and Liu scale	*OXT* gene, rs2740210, rs2770378 SNPs *AVP* gene, rs1887854, rs3787482, rs3761249 SNPs	*OXT* gene: no association between the Haplotype *OXT* rs2740210-rs2770378 and theory of mind was found. *AVP* gene: significant interaction between theory of mind and the haplotype rs1887854-rs3761249.
Lucht et al., 2013 [81]	76 healthy subjects Mean age: 19.45 years ± 2.31 RMET	*OXTR* gene, rs53576, rs2254298 e rs2228485 SNPs: T/T; T/C; C/C	*OXTR* gene: a significant result within rs2228485 SNP, the C/C genotype carriers show lower scores, on male face test images, than T genotype carriers, if the sex is analyzed separately the result seems to be significant only for girls. The rs53576 allele A carriers gave more correct answers in the face assessments of the task while rs2228485 SNP allele A carriers identified less frequently positive matrix images.
Kim et al., 2019 [82]	264 healthy Korean subjects Mean age: 20.8 years ± 2.5 Age range: 15–29 years TMPST	*OXTR* gene, rs1042778, rs237885, rs237887, rs2268490, rs4686301, rs2268493, rs2254298, rs13316193, rs53576 e rs2268498 SNPs	*OXTR*: there was no significant association between ToM scores and the variants of the *OXTR* gene.
Giralt-López et al., 2020 [83]	199 subjects: 38 patients with schizophrenia (Mean age: 24.92 years ± 3.90) 80 healthy first-degree relatives of schizophrenics (Mean age: 44.74 years ± 13.43) 81 healthy subjects unrelated to these families (Mean age: 34.77 years ± 12.53) HT; SPQ-CP	*OXTR* gene, rs53576 SNP: A allele carriers (A/A, A/G), G allele carriers (G/G)	*OXTR* gene: there was no association between ToM performance and rs53576 SNP; however, high scores in the SPQ-CP are related to worse performance on theory of mind G/G genotype.
Wade et al., 2014 [84]	300 healthy children Mean age: 4.79 ± 0.28 years Wellman and Liu Scale	*AVPR1A*, rs1042615, il rs7298346	*AVPR1A*: no association was found between theory of mind and rs1042615/rs7298346 polymorphisms
Reuter et al., 2020 [85]	435 healthy adults Age range: 18–72 years Mean age: 31.18 ± 13.13 years RMET	*MAO-A* uVNTR: low activity alleles carriers (2, 3 or 5 repeats); high activity alleles carriers (3.5 or 4 repeats)	*MAO-A* gene: carriers of low activity allele showed performances clearly inferior of who had the high activity allele
Startin et al., 2015 [86]	91 healthy men Age range: 18–40 years RMET	*EFHC2* gene, rs7055196 SNP: A allele carriers G allele carriers	*EFHC2* gene: A allele carriers showed better results than G allele carriers
Serrano-Juárez et al., 2021 [87]	26 subjects: 12 with Williams syndrome 7 with Down syndrome 7 healthy children Mean age: 11.73 years ± 3.75 Happé’s Strange Stories	*GTF2I* family genes: 1.1 Mb, 1.5 Mb e 1.8 Mb delections	*GTF2I* family genes: who was not carrying the *GTF2IRD1*, *GTF2I*, and *GTF2IRD2* genes had worse results than the healthy subjects.

Note: Reading the mind in the eyes task (RMET) [89]; Jerusalem Story Test of Interpersonal Understanding [91]; Wellman and Liu scale [92]; Representational theory of mind (RTM) [88]; ToM comic strips [93,94]; Hinting task (HT) [29]; White lie tasks [95]; Faux pas tasks [96]; Second-order false belief tasks [97]; Double bluff task [98]; List of Threatening Experiences (LTE-Q) [99,100]; False-belief contents [101]; False-belief location change task [102]; Theory of Mind Picture Sequencing Task (TMPST) [103,104]; Schizotypal Personality Questionnaire cognitive-perceptual (SPQ-CP) [90]; Happé’s Strange Stories [105].

## 4. Discussion

The overall aim of this review was to examine the role of genetic components on ToM tasks. Results indicate that several genes are associated with ToM in different age cohorts. With regard to studies carried out on children, the present systematic review shows an association with polymorphisms of *DRD4* gene [69,70], *OXTR* gene [78,80], *AVP* gene [79], *GTF2IRD1*, *GTF2I*, and *GTF2IRD2* genes [87]. These findings suggest that there might be a genetic correlation with ToM abilities that can be observed in typically developmental children [106]. Within adult populations, associations are shown with *DAT1* [72], *COMT* [76], *SCL6A4* [77], *OXTR* [81], *EFHC2* [86], and *MAO-A* [85]. These results show that the biological component still has an influence throughout life on ToM development. Notably, this latter consideration seems to be in line with previous research revealing that the genetic impact on empathy was higher in samples consisting of older participants [107].

There is evidence that ToM ability is related to the dopaminergic system [108]. Our findings add to the literature another proof of this link. In particular, we found an association with the *DRD4* gene, which plays a crucial role in the dopamine system [69]. In particular, the presence of the 7-repeat (7R) allele is linked to a decreased ability of dopamine to bind to receptors, resulting in reduced inhibition of postsynaptic neurons [109,110,111]. As a consequence, individuals carrying this allele exhibit heightened sensitivity to both negative and positive stimuli [112]. Another gene was also associated with the dopaminergic system: the *DAT1* gene, which is the candidate gene responsible for encoding the dopamine transporter (*DAT*), that participates in the reuptake of dopamine [70]. One study [72], focusing on *DAT1*, found that the group with 9/9 genotype showed a worse theory of mind performance; individuals carrying the DAT 9R allele have more *DAT* proteins available in the striatum compared to those who have two copies of the 10R allele, proposing reduced levels of dopamine in the striatum [113]. Since striatal dopaminergic activity is related to the ability to process emotional stimuli and motivational processes [114], our data indicate that individuals with the *DAT* 9R/9R gene variant may have a decreased ability to accurately interpret and understand the mental and emotional states of others [72]. Multiple genes have been identified to play a role in regulating the levels of dopamine in the synaptic space. One of these influential genes is catechol-O-methyltransferase (*COMT*), which is responsible for metabolizing dopamine [76]. The activity of *COMT* is influenced by genetic factors, and a common single nucleotide polymorphism (SNP) called Val158Met (rs4680) explains the majority of the observed variability in *COMT* activity [76]; the 158Val variant of the enzyme is associated with decreased enzymatic activity, which in turn causes an elevation in the levels of dopamine outside the cells [115]. The 158Val homozygote variant exhibits an enzyme activity that is approximately 35% lower than that of the 158Met homozygote variant in the human brain [116]. In a study, the Met allele showed significantly worse performance in theory of mind task than the Val allele group [60]. However, in the study conducted by Xia and colleagues [76], no association was found between the genotypes of the Val158Met (rs4680) SNP on the *COMT* gene and theory of mind (ToM) performance in adults. This result aligns with previous research conducted on typically developed children, which also showed no significant relationship between these genotypes and ToM abilities [70].

Concerning the *OXTR* gene, numerous studies acknowledge that genetic differences in the *OXTR* gene are linked to inclinations and behaviors that promote prosociality [80]. Moreover, research has demonstrated that intranasal administration of oxytocin can enhance trust levels [117,118]. It is plausible that the facilitation of social behavior and increased ingroup trust associated with oxytocin administration stem from its ability to enhance the recognition of emotional expressions in facial cues, as supported by studies conducted by Van Ijzendoorn and Bakermans-Kranenburg in 2012 [119].

Within the gene family of *GTF2I*, the results shown in the research [87] are in line with previous works, confirming that deletion (1.5 Mb) is associated with alterations in emotional processing and theory of mind [120]. Other studies have confirmed that direction: the deletion of *GTF2I* to *GTF2IRD2* is related to a deficit in social cognition [121,122]; therefore, it seems reasonable to state that the family gene of *GTF2I* is connected to mentalizing skills.

The only study with a positive correlation with *5-HTTLPR* shows that carriers of low-expression alleles (S/S; S/Lg; Lg/Lg) exhibited higher scores in recognizing negative mental states with increased exposure to negative life events [77]. This polymorphism shows that the presence of the short (S) allele in this genetic variation is associated with reduced transcriptional efficiency compared to the long (L) allele [123]. Consequently, individuals with the short allele have fewer functioning serotonin transporters, which affects the rate of serotonin neurotransmission and leads to variations in serotonin levels among individuals [123]. The result of the study [77] was mediated by negative life events; this is in line with the results of a recent systematic review where it was demonstrated that significant changes in the emotional and affective components of ToM are associated with PTSD [124]. However, the cognitive aspect of ToM has shown relatively less disruption, and in certain cases, it has even remained intact or unaffected [124].

On *MAO-A* genes, little is known about its role. One study investigating these genes’ role demonstrates that it does have a role in ToM. When the *MAO-A* gene is more active, it is believed to be associated with lower levels of serotonin (5-HT) [125]. So, when individuals possess low activity alleles of the *MAO-A uVNTR,* indicating higher 5-HT levels, they tend to exhibit better social cognition [85]. The role of the gene is also shown by another study [126] where the level of enzymatic activity of monoamine oxidase A (*MAO-A*), which is an indicator of serotonergic activity in the central nervous system, can be used to predict impairments in mentalizing abilities among individuals with schizophrenia. This association is particularly pronounced in individuals carrying the 4/4 genotype of the *MAO-A* VNTR polymorphism [126].

One study investigated the role of SNP rs7055196 in the *EFHC2* gene. This polymorphism has the potential to impact the process of gene transcription in the *EFHC2* gene and possibly influences other genes located nearby, such as *MAO-A* and *MAO-B*. The function of the *EFHC2* protein is not well understood [86]. However, its structure suggests a potential involvement in calcium binding. As a result, it could potentially influence various processes related to neuronal and intracellular signaling. These processes, in turn, play a role in the development of neural circuits associated with social cognition [86]; the SNP rs7055196 has the potential to affect the expression of the *MAO-A* and *MAO-B* genes which are involved in the metabolism of important neurotransmitters such as serotonin, noradrenaline, and dopamine [86]. Consequently, the influence of this SNP on the development of neural networks associated with social cognition may be independent and separate from its impact on these metabolic processes [127].

The *AVP* gene seems to have an association, which is in line with previous findings indicating that the administration of intranasal arginine vasopressin (*AVP*), not only influences social interaction [128] and emotional recognition [129], but it also affects the activity of the temporoparietal junction, a critical region involved in processing social information and performing theory of mind tasks [130]. In summary, these findings indicate that the administration of intranasal arginine vasopressin (*AVP*) can potentially influence social cognitive functioning, impacting both behavior and neural activity [131].

The present review guides future researchers to consider gender since there seem to be correlations between genes, gender, and performance in ToM tasks. In particular, two selected studies showed gender differences in this sociocognitive ability: females scored better at mentalizing others’ minds [76,78]. This outcome is in line with a recent meta-analysis [132] reporting that, on average, females tend to exhibit higher performance levels in tasks related to mentalizing or understanding the thoughts and feelings of others, as compared to males; the researchers explain this result as partially mediated by social experiences after birth, contributing to the observed differences [133]. However, a study [69] showed the opposite trend, reporting that the male group was better at mentalizing than the female group. Therefore, the debate is still open, and more research is needed to shed light on gender differences.

Another possible line that future research could further investigate is the relationship between haplotypes and ToM, as suggested by two included studies [78,79]. Studying haplotypes helps to identify the location of disease-causing genes by tracking recombination events in studies involving populations [134]. Therefore, it might help to understand deficits in ToM ability and clarify ToM roots more comprehensively. Future studies should also put an effort into recruiting elderly people because none of the selected studies considered this population. Moreover, the reason why gene polymorphisms are associated with ToM in some research and not in other research still needs to be clarified. The reasons for the inconsistent results might be due to the role of environmental factors; in fact, thanks to epigenetics, it is possible to study the impact of the environment on DNA expression. Epigenetics is known as the study of changes in gene activity not triggered by any mutation of the DNA sequence [135]; it discovers the environmental elements that contribute to affecting gene expression and, therefore, the development of a human being and their abilities such as understanding the mind of others [135]. Environmental experiences that affect an individual can cause what are called epigenomes, specific chemical tags that can silence (not express a protein) or trigger more actively specific genes [135]. These mutations may last for the entire life of a cell and can be transmitted for multiple generations [135]. These changes linked with certain genotypes can explain the different outcomes of the theory of mind development. For example, children with the short variant of DRD4 gene, in one of the research projects, seemed to score better than the long variant on the RTM [70], but on the other hand, in a study [136], the short variant appeared more vulnerable to the interrupted maternal communication with a disorganizational attachments, factors that can cause a worse ToM during development [135]. Overall, future studies should explore more in detail the mechanisms involved in this sociocognitive ability.

Given the complex interplay between risk factors involved in the development of ToM, future studies should implement the use of twin methodologies to clarify the role of genetics, particularly focusing on the same genes that have shown contradictory results, as well as shared and non-shared environmental risk factors. This approach with twin samples may help quantifying the extent of phenotypic variance explained by candidate genes and environment, enhancing our understanding of ToM etiology.

Our review helps lay the foundation for gaining insight into the influence of genetics on the ability to understand the minds of others. Following the biopsychosocial model, the development of any skill is the result of a synergistic interplay of three human components: the social, the psychological, and the biological/genetic [56]. Our goal was to understand which genes were associated with the healthy population to examine the biological component of mentalization, and the results appear to indicate a partial influence. When combined with other research on the developmental variables of the theory of mind (ToM), these results could create a comprehensive view of the origins of mentalization, encompassing social, psychological, and neuronal aspects that may be linked to genetic factors. Having a comprehensive understanding of the development of the theory of mind also allows us to identify risk factors for the etiology of the theory of mind and, consequently, genetic vulnerabilities in populations with a significant impairment of this ability.

This study delves into the influence of genetic polymorphisms on the capacity to comprehend the thoughts and emotions of others within the context of typical development. However, we can shed light on possible genetic risk factors associated with ToM dysfunction. For instance, in the case of a disorder characterized by severe ToM deficits, such as BPD [7], it is reasonable to assert that not only the environment plays a significant role, but biological factors also contribute. If a child is born with a genetic polymorphism that, on average, leads to a diminished capacity to infer the thoughts and emotions of others and is raised in an invalidating environment, it is plausible to expect the development of ToM impairments.

From a clinical perspective, there are numerous psychological interventions aimed at helping populations with difficulties in understanding the minds of others to enhance their ToM. However, these interventions often do not consider genetic factors. Understanding the role of the genetic component in target behaviors for intervention protocols or prevention strategies may have significant implications for designing prevention programs, determining program recipients, and comprehending individual variations in program effectiveness [137,138]. Several research studies have examined the genetic influence on resistance to interventions. For instance, Glenn and colleagues [139] investigated how genetic variants in the oxytocin receptor gene moderated the impact of coping power concerning the intervention’s delivery format. Results indicated that the gene variant influenced the effectiveness of the intervention based on group coping power versus individual coping power [139]. Awareness of these mechanisms will lead to more effective efforts in creating biologically informed, evidence-based prevention programs specifically targeted toward those who would benefit the most [140]. Furthermore, understanding the origin of such variation is crucial for enhancing existing preventive interventions and guiding the development of the next generation of interventions [140]. This understanding may also shed light on why certain ToM interventions can falter due to genetic differences or susceptibility. In conclusion, studying genes related to ToM performance in healthy subjects allows, on the one hand, the comprehension of genetic risk factors for the etiopathogenesis of ToM and, on the other hand, the understanding of how and why intervention measures may be influenced by genetic risks to improve and fine-tune such interventions [141].

## 5. Limits

For a better interpretation of the results presented, some limitations must be considered. First of all, the tests used to assess ToM were very heterogeneous. Moreover, only one article [76] investigated ToM by breaking it down into its two dimensions, affective and cognitive [1], while the remaining research considered the construct in its unity. This is an important limitation, as other types of conclusions might be drawn using different tests.

Moreover, the different ethnic backgrounds were not investigated enough in the existing scientific evidence. Genetic studies were susceptible to population stratification, and the unknown race of participants might limit the application of findings to different populations. Another limit is the number of participants; in fact, several studies used samples including less than 100 subjects.

Furthermore, the age of the subjects varied a lot, and some studies did not clearly distinguish adolescents and adults. Future research should include larger and more diverse populations in order to replicate the studies’ results.

Another important point concerns statistical analyses. The studies included different covariates, hampering the possibility of drawing homogeneous and linear results. Overall, it must be noted that the selected studies did not claim to outline a causal relationship but aimed at clarifying the correlation between ToM and polymorphic variations of genes.

There are many confounders when looking for ToM ability, for example, developmental stages, cognitive abilities, and social environments and others; the problem is always to understand how these elements relate to each other and with the genetic component. Therefore, longitudinal studies are particularly valuable for controlling confounders in research because they track the same subjects over an extended period. This repeated observation allows researchers to monitor changes and establish temporal sequences, which helps in distinguishing cause-and-effect relationships. By observing how these factors change over time in relation to ToM capabilities, researchers can more accurately assess the impact of each variable and reduce the likelihood of confounding bias.

A fundamental limitation is represented by the fact that the presented research takes into consideration restricted types of polymorphisms due to difficulties for scientists to recruit and analyze people with a wide variety of gene polymorphisms in interaction with ToM performance; however, this can leave behind important results on the influence of other genes on mentalizing.

## 6. Conclusions

Despite these limitations, the present systematic review provides partial support for the contribution of some genes to ToM performance in children and adults. Among the studies that examine preschool children, the ability to mentalize is found to be related to different genetic polymorphisms of genes: *DRD4* [69,70], *OXTR* [78,80], *AVP* [79] and *GTF2IRD1*, *GTF2I*, and *GTF2IRD2* [87]. Studies conducted on adults are more numerous, and they show stronger correlations with different polymorphisms of the following genes: *DAT1* [72], *COMT* [76], *SCL6A4* [77], *OXTR* [81], *EFHC2* [86], and *MAO-A* [85].

Since the present systematic review has revealed contrasting results within the same class of genes, showing a significant association in some studies but not in others, it is hard to draw any solid conclusion that clarifies the genes’ role in ToM development.

## Data Availability

No new data were created or analyzed in this study. Data sharing is not applicable to this article.

## References

[1] Brothers L., Ring B. (1992). A neuroethological framework for the representation of minds. J. Cogn. Neurosci..

[2] Kalbe E., Schlegel M., Sack A.T., Nowak D.A., Dafotakis M., Bangard C., Brand M., Shamay-Tsoory S., Onur O.A., Kessler J. (2010). Dissociating cognitive from affective theory of mind: A TMS study. Cortex.

[3] Cassetta B.D., Pexman P.M., Goghari V.M. (2018). Cognitive and Affective Theory of Mind and Relations with Executive Functioning in Middle Childhood. Merrill-Palmer Q..

[4] Longobardi E., Spataro P., D’Alessandro M., Cerutti R. (2017). Temperament Dimensions in Preschool Children: Links with Cognitive and Affective Theory of Mind. Early Educ. Dev..

[5] Gabriel E.T., Oberger R., Schmoeger M., Deckert M., Vockh S., Auff E., Willinger U. (2021). Cognitive and affective Theory of Mind in adolescence: Developmental aspects and associated neuropsychological variables. Psychol. Res..

[6] Premack D., Woodruff G. (1978). Does the chimpanzee have a theory of mind?. Behav. Brain Sci..

[7] Bateman A., Fonagy P. (2016). Mentalization-Based Treatment for Personality Disorders: A Practical Guide.

[8] Sloover M., van Est L.A., Janssen P.G., Hilbink M., van Ee E. (2022). A meta-analysis of mentalizing in anxiety disorders, obsessive-compulsive and related disorders, and trauma and stressor related disorders. J. Anxiety Disord..

[9] Szamburska-Lewandowska K., Konowałek Ł., Bryńska A. (2021). Theory of Mind deficits in childhood mental and neurodevelopmental disorders. Psychiatr. Pol..

[10] Gernsbacher M.A., Yergeau M. (2019). Empirical failures of the claim that autistic people lack a theory of mind. Arch. Sci. Psychol..

[11] Fletcher-Watson S., McConnell F., Manola E., McConachie H. (2014). Interventions based on the Theory of Mind cognitive model for autism spectrum disorder (ASD). Cochrane Database Syst. Rev..

[12] Dinishak J. (2013). Mindblindness: A troubling metaphor?. Ethics Neurodiversity.

[13] Ponnet K.S., Roeyers H., Buysse A., De Clercq A., Van Der Heyden E. (2004). Advanced Mind-Reading in Adults with Asperger Syndrome. Autism.

[14] Korkmaz B. (2011). Theory of mind and neurodevelopmental disorders of childhood. Pediatr. Res..

[15] Brüne M., Walden S., Edel M.A., Dimaggio G. (2016). Mentalization of complex emotions in borderline personality disorder: The impact of parenting and exposure to trauma on the performance in a novel cartoon-based task. Compr. Psychiatry.

[16] Fonagy P., Target M. (2001). Attaccamento e Funzione Riflessiva.

[17] Bateman A.W., Fonagy P. (2004). Mentalization-Based Treatment of BPD. J. Personal. Disord..

[18] Allen J.G., Fonagy P., Bateman A.W. (2008). Mentalizing in Clinical Practice.

[19] Bateman A., Fonagy P. (2008). 8-Year Follow-Up of Patients Treated for Borderline Personality Disorder: Mentalization-Based Treatment versus Treatment as Usual. Am. J. Psychiatry.

[20] Rossouw T.I., Fonagy P. (2012). Mentalization-based treatment for self-harm in adolescents: A randomized controlled trial. J. Am. Acad. Child. Adolesc. Psychiatry.

[21] McGauley G., Yakeley J., Williams A., Bateman A. (2011). Attachment, mentalization and antisocial personality disorder: The possible contribution of mentalization-based treatment. Eur. J. Psychother. Couns..

[22] Bora E., Walterfang M., Velakoulis D. (2015). Theory of mind in behavioural-variant frontotemporal dementia and Alzheimer’s disease: A meta-analysis. J. Neurol. Neurosurg. Psychiatry.

[23] Yu R.L., Wu R.M. (2013). Social brain dysfunctions in patients with Parkinson’s disease: A review of theory of mind studies. Transl. Neurodegener..

[24] Abu-Akel A., Shamay-Tsoory S.G. (2013). Characteristics of theory of mind impairments in schizophrenia. Social Cognition in Schizophrenia: From Evidence to Treatment.

[25] Bora E., Yucel M., Pantelis C. (2009). Theory of mind impairment in schizophrenia: Meta-analysis. Schizophr. Res..

[26] Brüne M. (2005). “Theory of mind” in schizophrenia: A review of the literature. Schizophr. Bull..

[27] Langdon R., Coltheart M., Ward P. (2006). Empathetic perspective-taking is impaired in schizophrenia: Evidence from a study of emotion attribution and theory of mind. Cognit. Neuropsychiatry.

[28] Sprong M., Schothorst P., Vos E., Hox J., Van Engeland H. (2007). Theory of mind in schizophrenia: Meta-analysis. Br. J. Psychiatry.

[29] Corcoran R., Mercer G., Frith C.D. (1995). Schizophrenia, symptomatology and social inference: Investigating “theory of mind” in people with schizophrenia. Schizophr. Res..

[30] Frith C.D. (2000). The cognitive neuropsychology of schizophrenia. Int. J. Psychol..

[31] Pickup G.J., Frith C.D. (2001). Theory of mind impairments in schizophrenia: Symptomatology, severity and specificity. Psychol. Med..

[32] Vass E., Fekete Z., Simon V., Simon L. (2018). Interventions for the treatment of theory of mind deficits in schizophrenia: Systematic literature review. Psychiatry Res..

[33] Bora E., Eryavuz A., Kayahan B., Sungu G., Veznedaroglu B. (2006). Social functioning, theory of mind and neurocognition in outpatients with schizophrenia; mental state decoding may be a better predictor of social functioning than mental state reasoning. Psychiatry Res..

[34] Brüne M., Brüne-Cohrs U. (2006). Theory of mind—Evolution, ontogeny, brain mechanisms and psychopathology. Neurosci. Biobehav. Rev..

[35] Couture S.M., Granholm E.L., Fish S.C. (2011). A path model investigation of neurocognition, theory of mind, social competence, negative symptoms and real-world functioning in schizophrenia. Schizophr. Res..

[36] Harrington L., Siegert R., McClure J. (2005). Theory of mind in schizophrenia: A critical review. Cognit. Neuropsychiatry.

[37] Roncone R., Falloon I.R., Mazza M., De Risio A., Pollice R., Necozione S., Morosini P., Casacchia M. (2002). Is theory of mind in schizophrenia more strongly associated with clinical and social functioning than with neurocognitive deficits?. Psychopathology.

[38] Rasmussen C., Wyper K., Talwar V. (2009). The relation between theory of mind and executive functions in children with fetal alcohol spectrum disorders. J. Popul. Ther. Clin. Pharmacol..

[39] Lindinger N.M., Malcolm-Smith S., Dodge N.C., Molteno C.D., Thomas K.G., Meintjes E.M., Jacobson J.L., Jacobson S.W. (2016). Theory of Mind in Children with Fetal Alcohol Spectrum Disorders. Alcohol. Clin. Exp. Res..

[40] Burke J.D., Loeber R., Birmaher B. (2002). Oppositional defiant disorder and conduct disorder: A review of the past 10 years, part II. J. Am. Acad. Child. Adolesc. Psychiatry.

[41] Coy K., Speltz M.L., DeKlyen M., Jones K. (2001). Social-cognitive processes in preschool boys with and without oppositional defiant disorder. J. Abnorm. Child. Psychol..

[42] Mary A., Slama H., Mousty P., Massat I., Capiau T., Drabs V., Peigneux P. (2016). Executive and attentional contributions to Theory of Mind deficit in attention deficit/hyperactivity disorder (ADHD). Child. Neuropsychol..

[43] Bora E., Pantelis C. (2016). Meta-analysis of social cognition in attention-deficit/hyperactivity disorder (ADHD): Comparison with healthy controls and autistic spectrum disorder. Psychol. Med..

[44] Pineda-Alhucema W., Aristizabal E., Escudero-Cabarcas J., Acosta-Lopez J.E., Vélez J.I. (2018). Executive function and theory of mind in children with ADHD: A systematic review. Neuropsychol. Rev..

[45] Eyuboglu D., Bolat N., Eyuboglu M. (2018). Empathy and theory of mind abilities of children with specific learning disorder (SLD). Psychiatry Clin. Psychopharmacol..

[46] Singh J., Arun P., Bajaj M.K. (2021). Theory of Mind and Executive Functions in Children with Attention Deficit Hyperactivity Disorder and Specific Learning Disorder. Indian J. Psychol. Med..

[47] Smogorzewska J., Szumski G., Grygiel P. (2018). Same or different? Theory of mind among children with and without disabilities. PLoS ONE.

[48] Yirmiya N., Erel O., Shaked M., Solomonica-Levi D. (1998). Meta-analyses comparing theory of mind abilities of individuals with autism, individuals with mental retardation, and normally developing individuals. Psychol. Bull..

[49] Hughes C., Cutting A.L. (1999). Nature, Nurture, and Individual Differences in Early Understanding of Mind. Psychol. Sci..

[50] Hughes C., Plomin R. (2000). Individual differences in early understanding of mind: Genes, non-shared environment and modularity. Evolution and the Human Mind: Modularity, Language, and Meta-Cognition.

[51] Leslie A.M., Friedman O., German T.P. (2004). Core mechanisms in ‘theory of mind. ’ Trends Cogn. Sci..

[52] Baron-Cohen S., Leslie A.M., Frith U. (1985). Does the autistic child have a “theory of mind”?. Cognition.

[53] Fodor J. (1987). Psychosemantics.

[54] Leslie A.M. (1987). Pretense and representation: The origins of “theory of mind”. Psychol. Rev..

[55] Leslie A.M. (1988). Some implications of pretense for mechanisms underlying the child’s theory of mind. Parts of This Chapter Are Based on Papers Presented to the International Conference on Developing Theories of Mind, University of Toronto, May 1986.

[56] Engel G.L. (1977). The Need for a New Medical Model: A Challenge for Biomedicine. Science.

[57] Kreppner J.M., O’Connor T.G., Dunn J., Andersen-Wood L., English and Romanian Adoptees (ERA) Study Team (1999). The pretend and social role play of children exposed to early severe deprivation. Br. J. Dev. Psychol..

[58] Yagmurlu B., Berument S.K., Celimli S. (2005). The role of institution and home contexts in theory of mind development. J. Appl. Dev. Psychol..

[59] Luke N., Banerjee R. (2013). Differentiated associations between childhood maltreatment experiences and social understanding: A meta-analysis and systematic review. Dev. Rev..

[60] Bassett A.S., Caluseriu O., Weksberg R., Young D.A., Chow E.W. (2007). Catechol-O-methyl transferase and expression of schizophrenia in 73 adults with 22q11 deletion syndrome. Biol. Psychiatry.

[61] Cornish K., Burack J.A., Rahman A., Munir F., Russo N., Grant C. (2005). Theory of mind deficits in children with fragile X syndrome. J. Intellect. Disabil. Res..

[62] Dennis M., Lockyer L., Lazenby A.L., Donnelly R.E., Wilkinson M., Schoonheyt W. (1999). Intelligence patterns among children with high-functioning autism, phenylketonuria, and childhood head injury. J. Autism Dev. Disord..

[63] Koenig K., Klin A., Schultz R. (2004). Deficits in social attribution ability in Prader–Willi syndrome. J. Autism Dev. Disord..

[64] Garrard P., Martin N.H., Giunti P., Cipolotti L. (2008). Cognitive and social cognitive functioning in spinocerebellar ataxia: A preliminary characterization. J. Neurol..

[65] Lawrence K., Jones A., Oreland L., Spektor D., Mandy W., Campbell R., Skuse D. (2007). The development of mental state attributions in women with X-monosomy, and the role of monoamine oxidase B in the sociocognitive phenotype. Cognition.

[66] Tager-Flusberg H., Sullivan K. (2000). A componential view of theory of mind: Evidence from Williams syndrome. Cognition.

[67] Ronald A., Viding E., Happé F., Plomin R. (2006). Individual differences in theory of mind ability in middle childhood and links with verbal ability and autistic traits: A twin study. Soc. Neurosci..

[68] Leslie A.M. (1994). ToMM, ToBy, and Agency: Core architecture and domain specificity. Mapping the Mind: Domain Specificity in Cognition and Culture.

[69] Ben-Israel S., Uzefovsky F., Ebstein R.P., Knafo-Noam A. (2015). Dopamine D4 receptor polymorphism and sex interact to predict children’s affective knowledge. Front. Psychol..

[70] Lackner C., Sabbagh M.A., Hallinan E., Liu X., Holden J.J.A. (2012). Dopamine receptor D4 gene variation predicts preschoolers’ developing theory of mind. Dev. Sci..

[71] Opitz T., Schuwerk T., Paulus M., Kloo D., Osterhaus C., Lesch K., Sodian B. (2021). No links between genetic variation and developing theory of mind: A preregistered replication attempt of candidate gene studies. Dev. Sci..

[72] Tadmor H., Levin M., Dadon T., Meiman M.E., Ajameeh A., Mazzawi H., Rigbi A., Kremer I., Golani I., Shamir A. (2016). Decoding emotion of the other differs among schizophrenia patients and schizoaffective patients: A pilot study. Schizophr. Res. Cogn..

[73] Zahavi A.Y., Sabbagh M.A., Washburn D., Mazurka R., Bagby R.M., Strauss J., Kennedy J.L., Ravindran A., Harkness K.L. (2016). Serotonin and dopamine gene variation and theory of mind decoding accuracy in major depression: A preliminary investigation. PLoS ONE.

[74] Walter H., Schnell K., Erk S., Arnold C., Kirsch P., Esslinger C., Mier D., Schmitgen M.M., Rietschel M., Witt S.H. (2011). Effects of a genome-wide supported psychosis risk variant on neural activation during a theory-of-mind task. Mol. Psychiatry.

[75] Hargreaves A., Morris D.W., Rose E., Fahey C., Moore S., Cummings E., Tropea D., Gill M., Corvin A., Donohoe G. (2012). ZNF804A and social cognition in patients with schizophrenia and healthy controls. Mol. Psychiatry.

[76] Xia H., Wu N., Su Y. (2012). Investigating the genetic basis of theory of mind (ToM): The role of catechol-O-methyltransferase (COMT) gene polymorphisms. PLoS ONE.

[77] Kruijt A.W., Putman P., Van Der Does W. (2014). The 5-HTTLPR polymorphism, early and recent life stress, and cognitive endophenotypes of depression. Cogn. Emot..

[78] Wade M., Hoffmann T.J., Jenkins J.M. (2015). Gene–environment interaction between the oxytocin receptor (OXTR) gene and parenting behaviour on children’s theory of mind. Soc. Cogn. Affect. Neurosci..

[79] Wade M., Hoffmann T.J., Knafo-Noam A., O’Connor T.G., Jenkins J.M. (2016). Oxytocin and vasopressin hormone genes in children’s externalizing problems: A cognitive endophenotype approach. Horm. Behav..

[80] Wu N., Su Y. (2015). Oxytocin Receptor Gene Relates to Theory of Mind and Prosocial Behavior in Children. J. Cogn. Dev..

[81] Lucht M.J., Barnow S., Sonnenfeld C., Ulrich I., Grabe H.J., Schroeder W., Völzke H., Freyberger H.J., John U., Herrmann F.H. (2013). Associations between the oxytocin receptor gene (OXTR) and “mind-reading” in humans—An exploratory study. Nord. J. Psychiatry.

[82] Kim H.W., Kang J.I., An S.K., Kim S.J. (2019). Oxytocin receptor gene variants are associated with emotion recognition and resilience, but not with false-belief reasoning performance in healthy young Korean volunteers. CNS Neurosci. Ther..

[83] Giralt-Lopez M., Miret S., Soler J., Campanera S., Parellada M., Fananas L., Fatjo-Vilas M. (2020). The role of schizotypal traits and the OXTR gene in theory of mind in schizophrenia: A family-based study. Eur. Psychiatry.

[84] Wade M., Hoffmann T.J., Jenkins J.M. (2014). Association between the arginine vasopressin receptor 1A (AVPR1A) gene and preschoolers’ executive functioning. Brain Cogn..

[85] Reuter M., Felten A., Zamoscik V., Bravo R., Ugartemendia L., Kirsch P., Rodriguez A.B., Plieger T. (2020). Genetic and epigenetic serotonergic markers predict the ability to recognize mental states. Physiol. Behav..

[86] Startin C.M., Fiorentini C., de Haan M., Skuse D.H. (2015). Variation in the X-linked EFHC2 gene is associated with social cognitive abilities in males. PLoS ONE.

[87] Serrano-Juárez C.A., Prieto-Corona B., Rodríguez-Camacho M., Venegas-Vega C.A., Yáñez-Téllez M.G., Silva-Pereyra J., Salgado-Ceballos H., Arias-Trejo N., Miranda M.A.D.L. (2021). An exploration of social cognition in children with different degrees of genetic deletion in Williams syndrome. J. Autism Dev. Disord..

[88] Wellman H.M., Carey S., Gleitman L., Newport E.L., Spelke E.S. (1990). The Child’s Theory of Mind.

[89] Baron-Cohen S., Wheelwright S., Hill J., Raste Y., Plumb I. (2001). The “Reading the Mind in the Eyes” Test revised version: A study with normal adults, and adults with Asperger syndrome or high-functioning autism. J. Child. Psychol. Psychiatry.

[90] Pisula E., Danielewicz D., Kawa R., Pisula W. (2015). Autism spectrum quotient, coping with stress and quality of life in a non-clinical sample—An exploratory report. Health Qual. Life Outcomes.

[91] Knafo A., Steinberg T., Goldner I. (2011). Children’s low affective perspective-taking ability is associated with low self-initiated pro-sociality. Emotion.

[92] Wellman H.M., Liu D. (2004). Scaling of Theory-of-Mind Tasks. Child. Dev..

[93] Walter H., Ciaramidaro A., Adenzato M., Vasic N., Ardito R.B., Erk S., Bara B.G. (2009). Dysfunction of the social brain in schizophrenia is modulated by intention type: An fMRI study. Soc. Cogn. Affect. Neurosci..

[94] Walter H., Adenzato M., Ciaramidaro A., Enrici I., Pia L., Bara B.G. (2004). Understanding intentions in social interaction: The role of the anterior paracingulate cortex. J. Cogn. Neurosci..

[95] Happe F.G.E. (1995). The Role of Age and Verbal Ability in the Theory of Mind Task Performance of Subjects with Autism. Child. Dev..

[96] Baron-Cohen S., O’Riordan M., Stone V., Jones R., Plaisted K. (1999). Recognition of faux pas by normally developing children and children with Asperger syndrome or high-functioning autism. J. Autism Dev. Disord..

[97] Astington J.W., Pelletier J., Homer B. (2002). Theory of mind and epistemological development: The relation between children’s second-order false-belief understanding and their ability to reason about evidence. New Ideas Psychol..

[98] Jolliffe T., Baron-Cohen S. (1999). The strange stories test: A replication with high-functioning adults with autism or Asperger syndrome. J. Autism Dev. Disord..

[99] Brugha T., Bebbington P., Tennant C., Hurry J. (1985). The List of Threatening Experiences: A subset of 12 life event categories with considerable long-term contextual threat. Psychol. Med..

[100] Brugha T.S., Cragg D. (1990). The List of Threatening Experiences: The reliability and validity of a brief life events questionnaire. Acta Psychiatr. Scand..

[101] Hogrefe G.J., Wimmer H., Perner J. (1986). Ignorance versus false belief: A developmental lag in attribution of epistemic states. Child Dev..

[102] Wimmer H., Perner J. (1983). Beliefs about beliefs: Representation and constraining function of wrong beliefs in young children’s understanding of deception. Cognition.

[103] Bömmer I., Brüne M. (2006). Social cognition in “pure” delusional disorder. Cogn. Neuropsychiatry.

[104] Brüne M., Brüne M., Ribbert H., Schiefenhövel W. (2003). Social Cognition and Behaviour in Schizophrenia. The Social Brain.

[105] Happé F.G.E. (1994). An advanced test of theory of mind: Understanding of story characters’ thoughts and feelings by able autistic, mentally handicapped, and normal children and adults. J. Autism Dev. Disord..

[106] Sabbagh M.A., Seamans E.L. (2008). Intergenerational transmission of theory-of-mind. Dev. Sci..

[107] Knafo A., Uzefovsky F. (2013). Variation in Empathy: The Interplay of Genetic and Environmental Factors. https://psycnet.apa.org/record/2013-07286-005.

[108] Abu-Akel A., Shamay-Tsoory S. (2011). Neuroanatomical and neurochemical bases of theory of mind. Neuropsychologia.

[109] Tol H.H., Wu C.M., Guan H.C., Ohara K., Bunzow J.R., Civelli O., Kennedy J., Seeman P., Niznik H.B., Jovanovic V. (1992). Multiple dopamine D4 receptor variants in the human population. Nature.

[110] Asghari V., Sanyal S., Buchwaldt S., Paterson A., Jovanovic V., Van Tol H.H.M. (1995). Modulation of Intracellular Cyclic AMP Levels by Different Human Dopamine D4 Receptor Variants. J. Neurochem..

[111] D’souza U.M., Russ C., Tahir E., Mill J., McGuffin P., Asherson P.J., Craig I.W. (2004). Functional effects of a tandem duplication polymorphism in the 5′ flanking region of the DRD4 gene. Biol. Psychiatry.

[112] Paquet C., Portella A.K., Moore S., Ma Y., Dagher A., Meaney M.J., Kennedy J.L., Levitan R.D., Silveira P.P., Dube L. (2021). Dopamine D4 receptor gene polymorphism (DRD4 VNTR) moderates real-world behavioural response to the food retail environment in children. BMC Public Health.

[113] van de Giessen E.M., de Win M.M., Tanck M.W., van den Brink W., Baas F., Booij J. (2009). Striatal dopamine transporter availability associated with polymorphisms in the dopamine transporter gene SLC6A3. J. Nucl. Med..

[114] Badgaiyan R.D. (2010). Dopamine is released in the striatum during human emotional processing. Neuroreport.

[115] Lachman H.M., Papolos D.F., Saito T., Yu Y.M., Szumlanski C.L., Weinshilboum R.M. (1996). Human catechol-O-methyltransferase pharmacogenetics: Description of a functional polymorphism and its potential application to neuropsychiatric disorders. Pharmacogenetics Genom..

[116] Chen J., Lipska B.K., Halim N., Ma Q.D., Matsumoto M., Melhem S., Kolachana B.S., Hyde T.M., Herman M.M., Apud J. (2004). Functional analysis of genetic variation in catechol-O-methyltransferase (COMT): Effects on mRNA, protein, and enzyme activity in postmortem human brain. Am. J. Hum. Genet..

[117] Kosfeld M., Heinrichs M., Zak P.J., Fischbacher U., Fehr E. (2005). Oxytocin increases trust in humans. Nature.

[118] Baumgartner T., Heinrichs M., Vonlanthen A., Fischbacher U., Fehr E. (2008). Oxytocin shapes the neural circuitry of trust and trust adaptation in humans. Neuron.

[119] Van IJzendoorn M.H., Bakermans-Kranenburg M.J. (2012). A sniff of trust: Meta-analysis of the effects of intranasal oxytocin administration on face recognition, trust to in-group, and trust to out-group. Psychoneuroendocrinology.

[120] Järvinen A., Korenberg J.R., Bellugi U. (2013). The social phenotype of Williams syndrome. Curr. Opin. Neurobiol..

[121] Karmiloff-Smith A., Broadbent H., Farran E.K., Longhi E., D’Souza D., Metcalfe K., Tassabehji M., Wu R., Senju A., Happe F. (2012). Social cognition in Williams syndrome: Genotype/phenotype insights from partial deletion patients. Front. Psychol..

[122] Porter M.A., Dobson-Stone C., Kwok J.B., Schofield P.R., Beckett W., Tassabehji M. (2012). A role for transcription factor GTF2IRD2 in executive function in Williams-Beuren syndrome. PLoS ONE.

[123] Conway C.C., Slavich G.M. (2017). Behavior genetics of prosocial behavior. Compassion.

[124] Couette M., Mouchabac S., Bourla A., Nuss P., Ferreri F. (2020). Social cognition in post-traumatic stress disorder: A systematic review. Br. J. Clin. Psychol..

[125] Shih J.C., Thompson R.F. (1999). Monoamine oxidase in neuropsychiatry and behavior. Am. J. Hum. Genet..

[126] Tylec A., Kucharska-Pietura K., Jeleniewicz W., Czernikiewicz A., Stryjecka-Zimmer M. (2010). Functional polymorphism of genes inactivating catecholamines and emotional deficits in paranoid schizophrenia. Psychiatr. Pol..

[127] Bortolato M., Godar S.C., Alzghoul L., Zhang J., Darling R.D., Simpson K.L., Bini V., Chen K., Wellman C.L., Lin R.C.S. (2013). Monoamine oxidase A and A/B knockout mice display autistic-like features. Int. J. Neuropsychopharmacol..

[128] Tabak B.A., Meyer M.L., Castle E., Dutcher J.M., Irwin M.R., Han J.H., Lieberman M.D., Eisenberger N.I. (2015). Vasopressin, but not oxytocin, increases empathic concern among individuals who received higher levels of paternal warmth: A randomized controlled trial. Psychoneuroendocrinology.

[129] Guastella A.J., Kenyon A.R., Alvares G.A., Carson D.S., Hickie I.B. (2010). Intranasal arginine vasopressin enhances the encoding of happy and angry faces in humans. Biol. Psychiatry.

[130] Zink C.F., Kempf L., Hakimi S., Rainey C.A., Stein J.L., Meyer-Lindenberg A. (2011). Vasopressin modulates social recognition-related activity in the left temporoparietal junction in humans. Transl. Psychiatry.

[131] Kenyon A.R., Alvares G.A., Hickie I.B., Guastella A.J. (2013). The effects of acute arginine vasopressin administration on social cognition in healthy males. J. Horm..

[132] Warrier V., Grasby K., Uzefovsky F., Toro R., Smith P., Chakrabarti B., Khadake J., Mawbey-Adamson E., Litterman N., Hottenga J.J. (2017). A meta-analysis of cognitive empathy: Heritability and correlates of the ‘Reading the Mind in the Eyes’ Test with psychiatric conditions, psychological traits and subcortical volumes. Eur. Neuropsychopharmacol..

[133] Varella M.A.C. (2018). The Biology and Evolution of the Three Psychological Tendencies to Anthropomorphize Biology and Evolution. Front. Psychol..

[134] Crawford D.C., Nickerson D.A. (2005). Definition and Clinical Importance of Haplotypes. Annu. Rev. Med..

[135] Westby C.E. (2014). Social neuroscience and theory of mind. Folia Phoniatr. Logop..

[136] Gervai J., Novak A., Lakatos K., Toth I., Danis I., Ronai Z., Nemoda Z., Sasvari-Szekely M., Bureau J.-F., Bronfman E. (2007). Infant genotype may moderate sensitivity to maternal affective communications: Attachment disorganization, quality of care, and the DRD4 polymorphism. Soc. Neurosci..

[137] Jaffee S.R., Price T.S. (2007). Gene–environment correlations: A review of the evidence and implications for prevention of mental illness. Mol. Psychiatry.

[138] Moffitt T.E., Caspi A., Rutter M. (2006). Measured Gene-Environment Interactions in Psychopathology: Concepts, Research Strategies, and Implications for Research, Intervention, and Public Understanding of Genetics. Perspect. Psychol. Sci..

[139] Glenn A.L., Lochman J.E., Dishion T., Powell N.P., Boxmeyer C., Qu L. (2018). Oxytocin receptor gene variant interacts with intervention delivery format in predicting intervention outcomes for youth with conduct problems. Prev. Sci..

[140] Musci R.J., Schlomer G. (2018). The Implications of Genetics for Prevention and Intervention Programming. Prev. Sci..

[141] Dick D.M. (2018). Commentary for special issue of prevention science “using genetics in prevention: Science fiction or science fact?”. Prev. Sci..

